# Hypertension: A common, yet poorly managed condition. What can we do better?

**DOI:** 10.1016/j.clinme.2026.100604

**Published:** 2026-06-04

**Authors:** Abilash Sathyanarayanan, Indranil Dasgupta, Ponnusamy Saravanan

**Affiliations:** Diabetes and Endocrinology, Nottingham University Hospitals NHS Trust, UK; University Hospitals Birmingham NHS Foundation Trust, UK; School of Health Sciences, University of Birmingham, UK; Warwick Applied Health, Warwick Medical School, University of Warwick, Coventry, UK; Centre for Global Health, University of Warwick, Coventry, UK; Centre for Early Life, University of Warwick, Coventry, UK; Diabetes, Endocrinology and Metabolism, George Eliot Hospital NHS Trust, Nuneaton, UK

Hypertension is a well-recognised risk factor for cardiovascular disease, and development and progression of chronic kidney disease (CKD). It is also the leading cause of premature death worldwide. Yet, diagnosis and management of hypertension continue to pose significant challenges. Fewer than 25% of patients with hypertension worldwide achieve adequate control of their blood pressure, and it remains a public health emergency.^1^ Managing a patient with high blood pressure is a routine occurrence in virtually every clinician’s practice. Despite being asymptomatic in most situations, the next steps in management can often be a fraught issue, especially when identified incidentally (eg unrelated clinic appointment). Therefore, each contact between a clinician and a patient with high blood pressure presents a valuable opportunity to reduce the risk of premature death and suffering.

In the latest issue of *Clinical Medicine*, my esteemed associate editors Prof Dasgupta and Dr Sathyanarayanan carefully commissioned topics that are hugely important for healthcare professionals across the world. Brief highlights of these articles are included here. Eltayeb *et al* provide a concise, evidence-based evaluation of the emerging role of wearable technologies – particularly, cuffless devices – in improving the detection and management of hypertension. Drawing on current scientific, engineering and clinical perspectives, they critically examine the potential utility and significant concerns regarding adoption of these in the clinical practice.^2^

Most patients with hypertension do not require referral to specialist services. However, clinical history, presentation and initial investigations provide important indicators for patients who may benefit from specialist review. Effective and comprehensive management of hypertension requires awareness of referral criteria and collaborative care from generalists and specialists.^3^ Two main indications for referral to a specialist hypertension service are suspected secondary hypertension and apparent resistance to antihypertensive treatment (defined as uncontrolled hypertension despite the use of optimum doses of three or more antihypertensive agents). Over half of the patients with apparent treatment resistance are non-adherent to prescribed medication. Jenkins *et al* outline a practical approach to recognising and assessing non-adherence using objective chemical adherence testing, and to managing medication non-adherence through non-judgemental, patient-centred discussion.^4^ Identifying non-adherence is vital, as failure to do so may lead to unnecessary treatment escalation, inappropriate investigations and avoidable healthcare costs, while addressing it can support and improve patient-centred outcomes.

Primary aldosteronism (PA), CKD and obstructive sleep apnoea (OSA) are three common forms of secondary hypertension. PA is a critical, yet frequently missed diagnosis. Phillips and Sathyanarayanan provide clear, evidence-based guidance on screening, focussig on contemporary interpretation of the test, practical advice on medication management, avoiding usual pitfalls, and when a specialist should be involved in the care.^5^ Afzal *et al* provide a practical, physician-focused update on the relationship between OSA and hypertension. It provides an overview of the epidemiology, pathophysiology, diagnosis and management of OSA-related hypertension.^6^

Doulton *et al* highlight the crucial importance of standardised BP measurement, lifestyle advice, achieving strict BP control, the use of renin-angiotensin system (RAS)-blocking drugs and adding a sodium-glucose co-transporter inhibitor type 2 in those with CKD; and the role of non-steroidal mineralocorticoid receptor antagonist in selected individuals. They also touch on emerging therapeutics, including aldosterone synthase and endothelin antagonists, and renal denervation.^7^

The availability of more than one first line class of antihypertensives and multiple drug choices in each class can make the decision of prescribing a drug complex. McNally *et al*^8^ provide a guide on how to choose the optimal antihypertensive therapy and achieve therapeutic benefits from it. They explore the common therapeutic pitfalls in making these decisions. Hypertension and heart failure exist together in many patients. Sundaram *et al*^9^ summarise the latest best practice guidance on managing high blood pressure in this situation. They particularly focus on safety- and tolerability-related decisions, which are pertinent in this patient population.

The issue also contains several original articles,^10^, ^11^, ^12^, ^13^, ^14^ topical review articles,^15^, ^16^, ^17^ a quality improvement work on the use of a non-invasive ventilation care bundle to reduce mortality in type 2 respiratory failure,^18^ a nationwide survey of directors of medical education on the barriers for medical training expansion in the UK,^19^ an interesting case report on parvovirus infection-associated fat embolism^20^ and letters to the editors that provide unique viewpoints. While they cover breadth of medicine and medical education, I pick the following three articles as my favourites for this issue. Murray *et al* argue that the word ‘nephrotoxic’ should not be used for RAS inhibitors.^17^ They make a strong case that in doing so, we can cause harm by avoiding them in people who actually need them the most. I think it is a must-read for all clinicians. Guru *et al* articulate the dilemma that most hospital-based clinicians face, ‘what to do to prevent venous thromboembolism (VTE) in stroke patients’, where the risk of both haemorrhagic transformation or expansion and VTE are the highest during the first 2 weeks following stroke.^16^ They provide a useful summary (and the discrepancy) of the current guidelines, highlight the need for urgent high-quality research evidence, and individualised approaches in the meantime. Finally, in the ever-rapidly increasing use of artificial intelligence (AI) in all walks of our life, Armstrong *et al* explored the role of generative AI (ChatGPT in this case) to improve the teaching of communication skills in breaking bad news.^10^ They show that AI-generated content can support and potentially improve compared to traditional training methods. They call for more research in this area and for a hybrid model that can integrate AI- and human-facilitated discussions to improve postgraduate medical education. Watch this space.

## Funding

This research did not receive any specific grant from funding agencies in the public, commercial, or not-for-profit sectors.

## Declaration of competing interest

Dr Sathyanarayanan and Prof Dasgupta are associate editors, and Prof Saravanan is the editor-in-chief of *Clinical Medicine*.
